# Supplementary material for: Study of the Materials and Techniques of a Rare Papier-Mâché Mushroom Model Crafted in H. Arnoldi Factory
Source: Molecules. 2023 Jan 20;28(3):1062. doi: 10.3390/molecules28031062 (PMC9919993; doi:10.3390/molecules28031062)
Supplement: Supplementary file 1 [file molecules-28-01062-s001.zip › molecules-2144817-supplementary.pdf]

molecules

Supplementary Materials

Study of the Materials and Techniques of a Rare Papier-mâché Mushroom Model Crafted in H. Arnoldi Factory

Supplementary Material

Table S1. Identification of paper pulp type of papier-mâché and labels used in the model, by Herzberg stain test.

**Sample**	**MO - Bright Field: Herzberg stain**	**Result interpretation** [15,22]
Papier mâché paste: largest mushroom's hat	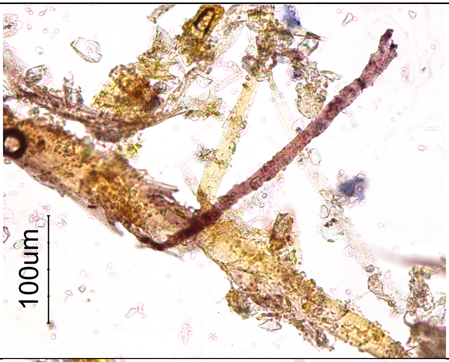	Groundwood pulp (bright yellow fibres)<br><br>Chemical pulp (purplish fibre)
Papier mâché paste: largest mushroom's stem	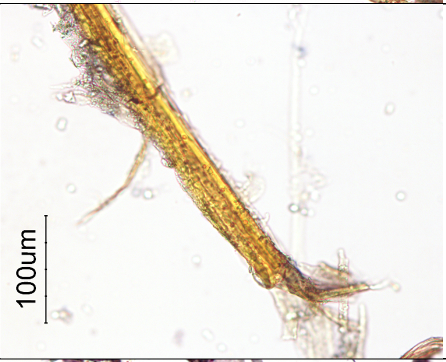	Groundwood pulp (bright yellow fibres)<br><br>Observation of tracheids from softwoods
Front label paper	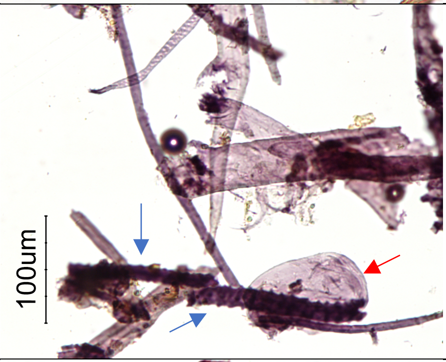	Chemical pulp (purplish fibres)<br><br>Straw pulp (bleached or unbleached): epidermal cells (blue arrows) and saclike parenchyma cells of wheat straw (red arrow)
Bottom label paper	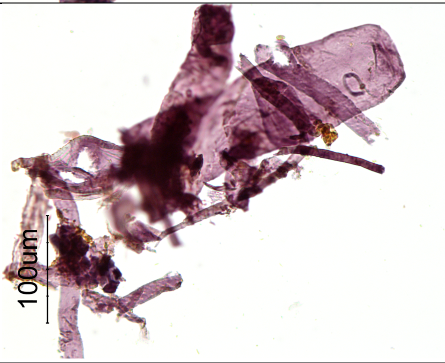	Chemical pulp (purplish fibres)
